# Epstein–Barr Virus-Mediated Apoptosis Evasion in Epithelial Malignancies: Molecular Mechanisms and Therapeutic Implications

**DOI:** 10.3390/biology15141121

**Published:** 2026-07-10

**Authors:** Rancés Blanco, Carmen Soto, Juan P. Muñoz

**Affiliations:** 1 Independent Researcher, Av. Vicuña Mackenna Poniente 6315, Santiago 8240000, Chile; rancesblanco1976@gmail.com; 2 Centro de Estudios de Proteínas, Facultad de Biología, Universidad de la Habana, La Habana 10400, Cuba; carmensoto@fbio.uh.cu; 3 Laboratorio de Bioquímica, Departamento de Química, Facultad de Ciencias, Universidad de Tarapacá, Arica 1000007, Chile

**Keywords:** Epstein–Barr virus, viral oncogenesis, apoptosis, latent proteins, lytic gene products, miR-BARTs, EBV-associated epithelial tumors, apoptosis-targeted therapy

## Abstract

Epstein–Barr virus (EBV) is a highly prevalent oncogenic virus that establishes persistent infection in most of the human population and is strongly associated with several lymphoid and epithelial malignancies, particularly nasopharyngeal carcinoma, gastric carcinoma, and lymphoepithelial carcinoma. This review summarizes current knowledge on the relationship between EBV infection and apoptosis regulation in epithelial cancers, with emphasis on how viral persistence may contribute to tumor cell survival and therapeutic resistance. The manuscript reviews evidence on EBV genome organization, latent and lytic infection programs, the epidemiology of EBV-associated epithelial tumors, and the main intrinsic and extrinsic apoptotic pathways. It then discusses how viral proteins, including latent membrane proteins, Epstein–Barr nuclear antigen 1 (EBNA1), BHRF1, and BARF1, as well as EBV-encoded microRNAs, modulate key apoptotic regulators such as p53, Bcl-2 family members, death receptor pathways, and caspases. Current evidence indicates that EBV can promote apoptosis resistance through coordinated effects on mitochondrial and death receptor-mediated cell death. Understanding these mechanisms may help clarify the contribution of EBV to epithelial oncogenesis and support therapeutic strategies aimed at restoring apoptotic sensitivity in EBV-associated tumors.

## Simple Summary

Epstein–Barr virus (EBV) infects most people worldwide and remains in the body for life. While infection is usually harmless, EBV is strongly linked to several cancers, including nasopharyngeal carcinoma, a subset of gastric cancers, and lymphoepithelial carcinoma. Patients with these tumors often experience treatment challenges because cancer cells can survive despite therapies designed to eliminate them. This review examines current evidence showing that EBV may contribute to this problem by helping infected tumor cells resist cell death. Understanding how EBV supports tumor survival is important because it may explain why some cancers are difficult to treat and may help identify new therapeutic opportunities. Emerging approaches that target EBV-related pathways or increase the sensitivity of tumor cells to treatment could improve outcomes for patients with EBV-associated cancers.

## 1. Introduction

The oncogenic viruses constitute a major contributor to cancer development worldwide, accounting for nearly 20% of the global cancer burden, although this proportion varies across populations and sociodemographic settings [1]. A shared feature among virus-associated malignancies is the capacity of viral genomes to persist long term within host cells, thereby creating conditions that support malignant transformation [2]. Among the recognized human oncogenic viruses, Epstein–Barr virus (EBV), a member of the *Herpesviridae* family, has emerged as a particularly important pathogen because of its high prevalence and broad oncogenic potential [3].

EBV is a double-stranded DNA virus first identified in 1964 that establishes persistent infection in approximately 95% of the human population [3]. Viral transmission occurs predominantly through nasopharyngeal secretions, and infection is primarily directed toward B lymphocytes, where EBV establishes lifelong latency [4,5]. EBV primary infection is usually asymptomatic during childhood [6]. However, acquisition of the virus during early adulthood frequently results in infectious mononucleosis, affecting nearly half of infected individuals [7,8]. The ability of EBV to maintain persistent infection is considered a fundamental determinant of its oncogenic activity and plays a central role in virus-associated carcinogenesis [9,10].

The oncogenic spectrum of EBV encompasses both lymphoid and epithelial malignancies. Strong associations have been established with Burkitt lymphoma, Hodgkin’s lymphoma, extranodal natural killer/T-cell lymphoma, and other lymphoproliferative disorders [11]. In epithelial tissues, EBV is recognized as a major etiological factor in nasopharyngeal carcinoma (NPC) and in a subset of gastric carcinomas [12,13]. Although EBV DNA and viral proteins have been detected in oral, breast, and cervical carcinomas, current evidence remains insufficient to establish a definitive causal relationship between EBV infection and the development of these tumors [14,15,16].

Long-term persistence of EBV depends on the virus’s ability to evade immune surveillance and preserve the viability of infected cells. Among the mechanisms involved, suppression of apoptosis represents a critical strategy [17,18]. EBV encodes multiple proteins and non-coding RNAs capable of modulating apoptotic signaling pathways. Latent membrane protein 1 (LMP1), which functionally resembles the CD40 receptor, activates pro-survival pathways such as NF-κB, PI3K/Akt, and MAPK, thereby promoting resistance to programmed cell death [19]. Likewise, Epstein–Barr nuclear antigen 1 (EBNA1), beyond its role in episome maintenance, can indirectly interfere with apoptosis through alterations in host gene expression and impairment of antigen presentation [20]. EBV-derived microRNAs, particularly those encoded within the BART region, can reinforce this survival phenotype by targeting immune regulators and pro-apoptotic molecules such as PUMA, BIM, and caspase-3, which may attenuate intrinsic apoptotic signaling [21,22].

Apoptosis resistance not only facilitates viral persistence but also contributes to the initiation and progression of EBV-associated cancers [23]. Because many conventional therapies, including chemotherapy and radiotherapy, rely on apoptosis induction to eradicate malignant cells, impairment of these pathways can compromise treatment efficacy [24,25,26]. Consequently, EBV-positive tumors are frequently associated with inferior therapeutic responses and a greater risk of recurrence [27,28]. These observations have stimulated the development of therapeutic strategies aimed at restoring apoptotic competence, including BH3 mimetics, immune checkpoint inhibitors, and agents directed against viral gene products [29]. Therefore, a detailed understanding of how EBV manipulates apoptotic pathways is essential for clarifying mechanisms of viral oncogenesis and identifying novel therapeutic opportunities in EBV-associated malignancies.

## 2. EBV Genome Organization and Replication

EBV is a linear double-stranded DNA virus with a genome of approximately 172 kilobase pairs enclosed within an icosahedral nucleocapsid and surrounded by a lipid envelope containing multiple viral glycoproteins [30,31]. EBV exhibits dual tropism and primarily infects B lymphocytes and epithelial cells through distinct entry mechanisms. In B cells, viral attachment is initiated by binding of the major envelope glycoprotein gp350 to CD21 or CD35, while interaction of gp42 with MHC class II molecules facilitates membrane fusion [32,33]. In epithelial cells, entry is mainly mediated by the gH/gL glycoprotein complex through interactions with integrins including αvβ6, αvβ8, and αvβ5 [34,35]. Additional entry factors have also been described. The viral glycoprotein BMRF2 enhances infection of polarized epithelial cells through interactions with α3, α5, and β1 integrins, promoting entry through the basolateral membrane [36], while the ephrin receptor EphA2 has likewise been implicated as an epithelial entry mediator [37].

**Figure 1:**
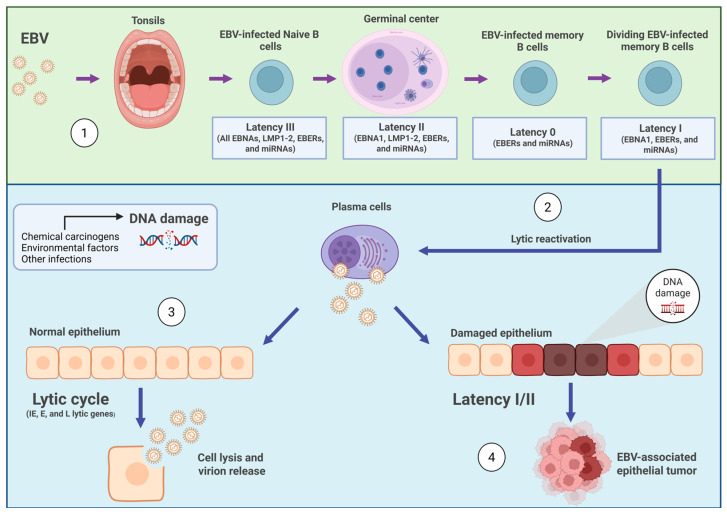
Overview of the EBV life cycle and its relationship to epithelial carcinogenesis. (1) EBV infects naïve B cells and establishes distinct latency programs during B-cell differentiation, progressing from latency III to latency 0 in memory B cells. (2) Differentiation of infected memory B cells into plasma cells can trigger lytic reactivation and virion production. (3) Released virions infect epithelial cells, where productive lytic replication results in cell lysis and viral dissemination. (4) Infection of damaged epithelium may favor latent infection (latency I/II), contributing to the development of EBV-associated epithelial malignancies. Created in BioRender. Muñoz, J.P. (2026) https://BioRender.com/kop3gsv.

Following receptor engagement and membrane fusion, the viral genome is delivered to the nucleus, where EBV establishes either a latent or lytic program depending on the infected cell type and its differentiation state. In B-cells, EBV infection is predominantly latent, promoting long-term persistence through episomal maintenance and restricted viral gene expression, while periodic lytic reactivation enables viral dissemination. In contrast, normal epithelial cells more commonly support lytic replication and virion release, whereas latent infection appears to occur at very low frequencies in nasopharyngeal and oral epithelia [38,39]. This balance between latency and lytic replication is regulated by both viral and host factors and allows EBV to alternate between persistence and dissemination according to the cellular and microenvironmental context (Figure 1).

Progress in understanding EBV infection in epithelial cells has historically been limited by the lack of suitable experimental systems; however, organotypic epithelial culture models have provided evidence that EBV can replicate within stratified epithelia without requiring cells expressing exclusively latent gene programs [40,41,42].

The transition from latency to lytic replication depends on activation of the immediate-early (IE) genes *BZLF1* and *BRLF1*, which encode the transcriptional regulators Zta and Rta, respectively [43,44]. Their expression, controlled through the *Zp* and *Rp* promoters, initiates the lytic transcriptional cascade and is essential for downstream early gene activation [45]. Reactivation may be influenced by epithelial differentiation, with Blimp1 implicated in Zp activation [46], and can also be induced by chemical stimuli such as TPA, sodium butyrate, and calcium ionophores, whereas DNA methylation and histone deacetylation suppress IE gene expression [47].

Early lytic genes encode proteins required for viral DNA replication, including the viral DNA polymerase BALF5 [48], the processivity factor BMRF1 [49], and components of the helicase–primase complex such as BBLF4, BSLF1, and BBLF2/3 [50]. BMRF1 and BRRF1 additionally participate in activation of the lytic origin of replication (*oriLyt*) [51]. Viral DNA synthesis proceeds through a rolling-circle mechanism that generates concatemeric intermediates subsequently cleaved and packaged into progeny virions [52,53]. Following genome replication, late lytic genes are expressed and predominantly encode structural components, including nucleocapsid proteins and envelope glycoproteins such as gp350/220, gp85, gp42, and gp25. In epithelial tissues, productive replication and virion assembly are typically restricted to the upper differentiated layers of stratified epithelia [42], facilitating viral dissemination to neighboring cells [54].

In contrast, EBV latency is characterized by maintenance of the viral genome as a circular episome within the nucleus of memory B-cells together with restricted viral gene expression, thereby contributing to immune evasion [55,56]. The latent transcriptional repertoire includes the *EBNA1*, *EBNA2*, *EBNA3A-3C*, and *EBNALP*, *LMP1*, *LMP2A*, and *LMP2B*, and the non-coding *EBER* transcripts [32,57,58].

EBV also encodes approximately 44 mature microRNAs derived from the *BHRF1* and *BART* regions of the viral genome [57,58]. While *BHRF1* miRNAs are preferentially expressed during the lytic cycle and latency III programs, *BART* miRNAs are abundantly expressed in latently infected epithelial and carcinoma cells, including nasopharyngeal and gastric carcinoma-derived cells [59,60]. These viral miRNAs target host immune regulators, pro-apoptotic molecules, and viral transcripts, thereby modulating the cellular environment in ways that favor viral persistence and tumorigenesis [61,62]. Together, the capacity of EBV to alternate between latent and lytic programs, combined with its restricted and highly regulated gene expression profile, provides a molecular framework through which the virus can influence host–cell survival and apoptosis.

## 3. Epidemiology of EBV Infection in Epithelial Cancers

While EBV is an established etiological agent in NPC and a subset of gastric carcinomas and is strongly and consistently associated with lymphoepithelial carcinoma (LEC), its involvement in other epithelial tumors remains less clearly defined. For oral, cervical, breast, lung, colorectal, and prostate cancers, the available evidence indicates a potential association rather than proof of causality. Therefore, the detection of EBV DNA, RNA, or viral proteins in tumor tissues should not be interpreted as definitive evidence of a causal role in carcinogenesis. The following sections critically examine the current evidence regarding the presence of EBV in these malignancies as well as its potential contribution to tumor development and progression.

### 3.1. Nasopharyngeal Carcinoma

NPC is the predominant histological type of nasopharyngeal cancer. In 2022, an estimated 120,416 new cases and 73,476 deaths from nasopharyngeal cancer were reported worldwide [63]. Projections indicate that, by 2040, the annual global burden will increase to 179,476 cases and 113,851 deaths [64]. NPC exhibits a distinctive geographic and ethnic distribution, with the highest incidence observed in Southern China, where rates approach 20 cases per 100,000 individuals. Intermediate incidence rates have been reported in Southeast Asia and Northern Africa (5–15 per 100,000), whereas most other regions of the world show a low incidence, generally below 1 case per 100,000 population [65,66]. Interestingly, endemic areas typically exhibit a unimodal distribution with a peak incidence between 50 and 60 years of age, whereas non-endemic regions show a bimodal pattern, with an initial peak between 15 and 24 years and a second peak between 65 and 79 years of age [67,68].

NPC development is associated with a multifactorial etiology involving environmental, lifestyle, and genetic factors. Consumption of salted fish, tobacco smoking, alcohol intake, exposure to air pollutants such as NO_2_, and host genetic susceptibility have all been associated with increased NPC risk [69,70]. Among these factors, EBV is recognized as a well-established etiological agent in NPC pathogenesis [12].

The prevalence of EBV-positive NPC varies according to geographic region and histological subtype. In low-risk populations, including Caucasian individuals from North America and Western countries, EBV has been detected in 47.0–83.2% of NPC cases, depending on the study population and tumor histology [71,72,73]. In endemic regions, EBV is detected in more than 97.0% of non-keratinizing undifferentiated NPC cases [74,75,76,77], while studies from non-endemic regions such as Brazil and the Netherlands also reported high EBV positivity rates in the same histological subtype [72,78]. These observations highlight the strong association between EBV infection and NPC, particularly in non-keratinizing tumors, supporting the relevance of this malignancy as a model for investigating EBV-mediated mechanisms of apoptosis regulation and tumor survival.

### 3.2. Gastric Carcinoma

Gastric cancer (GC) is the fifth most commonly diagnosed cancer and the fifth leading cause of cancer-related mortality worldwide, with 968,350 new cases and approximately 659,853 deaths reported in 2022 [63]. Its incidence shows marked geographic variation, with the highest rates observed in Eastern and Western Asia, Eastern and Southern Europe, and South America, where incidence ranges from 10.4 to 32.1 cases per 100,000 of the population. In contrast, lower incidence rates (<6.4 per 100,000) have been reported in Africa, North America, Northern Europe, Australia, and New Zealand [79]. The global burden of GC is expected to increase substantially over the coming years, reaching an estimated 1.8 million new cases and 1.3 million deaths annually by 2040 [80].

Gastric carcinogenesis is a multifactorial process influenced by environmental, infectious, and host-related factors. Infection with *Helicobacter pylori* (*H. pylori*) is recognized as a major etiological factor in GC development [81,82]. Persistent *H. pylori* infection, alone or in combination with additional risk factors, has been associated with gastric ulcer formation and increased GC risk [81]. Other factors implicated in GC include high salt intake, excessive consumption of smoked meat, smoking, alcohol abuse, genetic predisposition, and viral infections. In this context, both HPV and EBV have been proposed to cooperate in GC development [83,84].

The association between EBV and GC has been documented worldwide. In a meta-analysis by Dokanei et al. (2024), which included 87 studies and 30,242 GC cases, the global prevalence of EBV-positive tumors was estimated at 9.5% (95% CI: 8.2–11.0) [85]. Subgroup analyses revealed substantial geographic variability, with prevalence rates ranging from 2.8% in Canada to 22.7% in Algeria. Similarly, individual studies reported frequencies ranging from 2.1% in Iran and 3.4–4.5% in China to 22% in southern Chile [86,87,88,89]. Although another meta-analysis suggested that the prevalence of EBV in conventional GC is broadly comparable across geographic regions [90], the observed heterogeneity indicates that regional and environmental factors, including coinfections, dietary patterns, lifestyle exposures, and population-specific characteristics, may influence the prevalence of EBVaGC. Nevertheless, the consistent detection of EBV in a subset of GC supports the relevance of EBV-associated molecular mechanisms, including apoptosis dysregulation, in gastric tumorigenesis. In EBVaGC, most cases are diagnosed in older adults, with some studies reporting affected patients predominantly between 50 and 75 years of age [87,91,92].

### 3.3. LEC

LEC, also referred to as lymphoepithelioma-like carcinoma, is a rare and high-grade malignancy characterized by undifferentiated carcinoma accompanied by dense non-neoplastic lymphoplasmacytic infiltrates [93]. Histologically, LEC is classified as a non-keratinizing squamous cell carcinoma and shares morphological features with undifferentiated NPC, although it arises outside the nasopharynx [93,94]. LEC occurs predominantly in the head and neck region, including the paranasal sinuses, oral cavity, and larynx, and is most frequently reported in the salivary glands [94,95,96,97]. Similar tumors have also been described in other epithelial organs, including the stomach, lung, skin, breast, liver, esophagus, uterine cervix, and prostate [98,99,100,101,102,103,104,105].

A strong association between EBV infection and LEC has been consistently documented across multiple anatomical sites. In a systematic review by Rytkönen et al. comprising 112 head and neck LEC cases from 37 studies, EBV was detected in 87.5% of tested tumors [106]. Likewise, a meta-analysis by Mozaffari et al. reported EBV positivity in 96.1% (99/103) of salivary gland LEC cases [107]. EBV expression was significantly more frequent in major salivary gland LEC compared with normal tissue and large-cell undifferentiated carcinomas, in which EBV was not detected [108].

High EBV prevalence has also been reported in extra-salivary LEC. In pulmonary LEC, EBV was detected in 93.8% (30/32) and 80.4% (37/46) of cases, with significantly lower detection rates observed in non-LEC lung tumors [109]. Similarly, in gastric LEC, a meta-analysis by Lee et al. identified EBV in 86.4% (152/176) of tumors, representing a markedly higher prevalence than that observed in non-LEC GC (OR = 80.881; 95% CI: 49.6–131.9; *p* < 0.001) [110]. Consistent with these findings, a recent study estimated a global EBV prevalence of 75.9% (95% CI: 62.8–85.5) in gastric LEC, which remained significantly higher than in conventional gastric adenocarcinoma (*p* < 0.001) [90].

Although the reported prevalence estimates should be interpreted in light of substantial heterogeneity across studies, including differences in geographic origin, anatomical site, sample size, and EBV detection methods, the consistently high frequency of EBV positivity observed across independent cohorts supports a strong association between EBV and LEC.

Together, these observations demonstrate a strong and recurrent association between EBV infection and LEC across multiple epithelial sites, supporting the relevance of this malignancy as an additional model for investigating EBV-associated epithelial oncogenesis and viral mechanisms involved in tumor cell survival.

### 3.4. EBV Infection in Other Epithelial Tumors

EBV has also been detected in several additional epithelial malignancies that contribute substantially to global cancer incidence and mortality, including oral, cervical, breast, colorectal, lung, and prostate cancers [63]. However, unlike NPC, gastric carcinoma, and LEC, a definitive causal role for EBV in these tumors has not been established.

Evidence from pooled analyses nevertheless suggests a consistent epidemiological association between EBV detection and several epithelial malignancies. In oral squamous cell carcinoma (OSCC), a meta-analysis by She et al. reviewing 13 studies published between 1995 and 2016 reported that EBV positivity was associated with significantly higher odds of OSCC (pooled OR = 5.03; 95% CI: 1.80–14.01), although these case–control data cannot establish causality [111]. Similar findings were obtained in an independent meta-analysis, which estimated a 2.5-fold increased likelihood of OSCC among EBV-positive individuals (OR = 2.5; 95% CI: 1.2–5.36) [112].

Comparable associations have been described in cervical carcinoma. A meta-analysis by de Lima et al. including 25 studies and 2499 cervical samples reported pooled EBV prevalence rates of 27.34%, 34.67%, and 43.63% in low-grade squamous intraepithelial lesions (LSIL), high-grade squamous intraepithelial lesions (HSILs), and cervical carcinoma, respectively, all exceeding the prevalence observed in healthy controls (19.0%). In this pooled analysis, EBV detection was associated with higher odds of cervical cancer (OR = 4.01; 95% CI: 1.87–8.5; *p* < 0.001), an association that does not by itself establish causality [113].

Associations between EBV and additional epithelial tumors have also been reported. In breast cancer, pooled analyses estimated an EBV prevalence of 26.37% (95% CI: 22–31%) based on 44 studies, while a meta-analysis of 30 case–control studies identified a significant association between EBV infection and breast cancer risk (OR = 4.74; 95% CI: 2.92–7.69; *p* < 0.0001) [114]. In colorectal carcinoma, a meta-analysis involving 1954 patients reported a pooled EBV prevalence of 18% (95% CI: 12–26%) together with an increased risk estimate for colorectal cancer (OR = 3.4; 95% CI: 1.13–10.27) [115].

**Figure 2:**
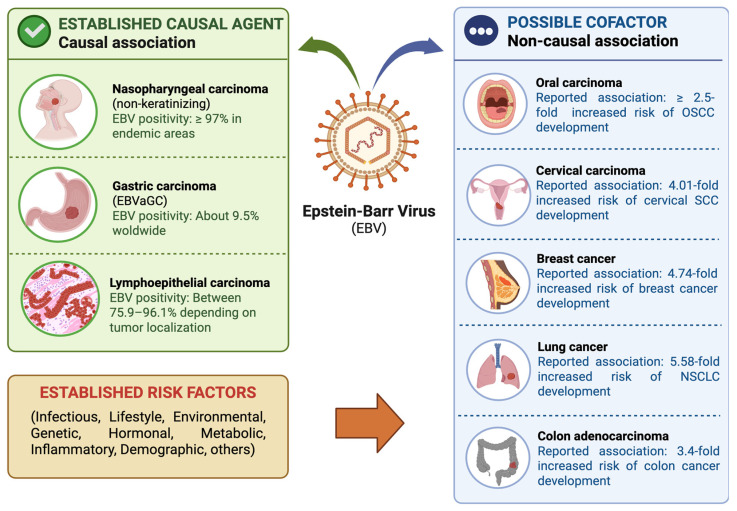
Association of EBV with epithelial malignancies. EBV is an established causal agent in non-keratinizing NPC and EBVaGC and is strongly associated with lymphoepithelial carcinoma (LEC), whereas its detection in oral carcinoma, cervical carcinoma, breast cancer, lung cancer, and colon adenocarcinoma suggests a possible cofactor role rather than a definitive causal association. In these malignancies, EBV may cooperate with established genetic, environmental, infectious, and lifestyle-related risk factors to promote tumor development. Created in BioRender. Munoz, J.P. (2026) https://BioRender.com/mp7gwnz.

Evidence linking EBV to lung cancer has likewise emerged from pooled analyses. Chen et al. reported EBV prevalence rates of 21.17% in small-cell lung cancer (SCLC) and 32.43% in non-small-cell lung cancer (NSCLC), together with a strong overall association between EBV infection and lung cancer risk (OR = 8.30; 95% CI: 4.08–16.9; *p* < 0.001) [116]. Risk estimates were higher for NSCLC (OR = 5.58; 95% CI: 1.98–15.72; *p* < 0.001) than for SCLC (OR = 2.13; 95% CI: 0.44–10.26; *p* = 0.001) [116]. In prostate cancer, EBV has also been detected in tumor tissues, with reported prevalence ranging from 37% to 49.6% [117,118,119]. These observations show the need for additional mechanistic studies to clarify whether EBV contributes to epithelial tumorigenesis through pathways such as viral-mediated regulation of cell survival and apoptosis (Figure 2).

The expression of some EBV lytic and latent genes detected in epithelial tumors is summarized in Table 1.

Together, these studies demonstrate recurrent detection of EBV across multiple epithelial malignancies and support epidemiological associations with several tumor types. Nevertheless, unlike NPC, GC, and LEC, the oncogenic contribution of EBV in these cancers remains incompletely defined. These observations underscore the need for additional mechanistic studies to clarify whether EBV contributes to epithelial tumorigenesis through pathways such as viral-mediated regulation of cell survival and apoptosis.

## 4. Apoptotic Pathways Relevant to EBV-Associated Epithelial Tumors

Apoptosis is a tightly regulated cellular process that preserves tissue homeostasis through the elimination of damaged, infected, or transformed cells. During apoptosis, cells undergo characteristic morphological changes, including cell shrinkage, chromatin condensation, DNA fragmentation, and plasma membrane blebbing, ultimately leading to the formation of apoptotic bodies [155]. Apoptotic signaling proceeds through two major pathways, the intrinsic (mitochondrial) and extrinsic (death receptor-mediated) pathways, which converge on caspase activation and culminate in apoptotic cell death [155,156]. Given its role in antiviral defense and tumor suppression, apoptosis represents a major barrier to viral persistence and tumor development. In EBV-associated epithelial malignancies, viral proteins and microRNAs predominantly target key regulatory nodes within both pathways.

The intrinsic apoptotic pathway is activated in response to intracellular stress, including DNA damage, oxidative stress, oncogene activation, and other disruptions of cellular homeostasis [157]. A central regulator of this pathway is the tumor suppressor p53, which promotes the expression of pro-apoptotic genes following cellular stress [158]. Mitochondrial apoptosis is primarily controlled by the Bcl-2 family, which comprises anti-apoptotic proteins, including Bcl-2, Bcl-xL, and Mcl-1, and pro-apoptotic proteins such as Bax, Bak, Bim, Puma, Bid, Bad, and Noxa [159]. Activation of Bax and Bak induces mitochondrial outer membrane permeabilization (MOMP), resulting in cytochrome c release and subsequent activation of caspase-9 and downstream executioner caspases [160]. Accordingly, p53 signaling, Bcl-2 family proteins, and MOMP constitute major regulatory nodes targeted by EBV to suppress apoptosis and promote infected-cell survival.

The extrinsic apoptotic pathway is initiated through the binding of the Fas ligand (FasL), tumor necrosis factor-α (TNF-α), or TNF-related apoptosis-inducing ligand (TRAIL) to their corresponding death receptors [156,161]. Receptor activation promotes assembly of the death-inducing signaling complex (DISC), leading to activation of caspase-8 and subsequent cleavage of executioner caspases, including caspase-3 and caspase-7 [162]. Extrinsic and intrinsic pathways are functionally interconnected through the BH3-only protein Bid. Following activation, caspase-8 cleaves Bid to generate truncated Bid (tBid), which promotes Bax/Bak-mediated mitochondrial permeabilization and amplifies apoptotic signaling [163,164]. Consequently, death receptor signaling, DISC assembly, caspase-8 activation, and Bid-dependent crosstalk with the mitochondrial pathway represent additional apoptotic checkpoints susceptible to EBV-mediated regulation.

The following section examines how EBV latent proteins, lytic gene products, and BamHI-A rightward transcripts (miR-BARTs) modulate these apoptotic nodes to promote infected-cell survival, facilitate viral persistence, and contribute to apoptosis resistance in EBV-associated epithelial malignancies.

## 5. EBV Products and Evasion of Apoptosis

EBV employs multiple viral proteins and microRNAs to modulate host apoptotic pathways and promote the survival of infected cells. Suppression of apoptosis contributes to viral persistence, maintenance of latency, and oncogenic transformation. This section summarizes the role of selected EBV-encoded proteins and microRNAs involved in apoptosis resistance in epithelial cells.

### 5.1. EBV-Encoded Proteins and Apoptosis Protection

#### 5.1.1. LMP1 and LMP2

Latent membrane proteins encoded by EBV are important mediators of apoptosis resistance and oncogenesis in infected epithelial cells. Among them, LMP1 is the best-characterized EBV oncoprotein and functions as a constitutively active mimic of the tumor necrosis factor receptor (TNFR) family member CD40 [165]. In lymphoid cells, LMP1 promotes resistance to apoptosis through activation of multiple pro-survival signaling pathways, including NF-κB, JAK/STAT, and PI3K/AKT, and by inducing the expression of anti-apoptotic proteins such as Bcl-2, Mcl-1, and Bfl-1 [19]. Together, these mechanisms protect EBV-infected cells from both intrinsic and extrinsic apoptotic stimuli. In addition, LMP1 interferes with death receptor-mediated apoptosis by downregulating Fas expression and disrupting death-inducing signaling complex (DISC) formation [166].

Additional evidence supports the role of LMP1 in suppressing pro-apoptotic signaling in epithelial malignancies. In NPC cells, LMP1 reduced the activity of the pro-apoptotic factor prostate apoptosis response-4 (Par-4) through activation of the PI3K/Akt pathway and increased Bcl-2 expression [167]. LMP1 has also been shown to upregulate survivin expression in NPC cells through p53 activation. However, despite activation of p53 signaling, this response favored G1/S cell-cycle progression rather than apoptosis induction [168]. Furthermore, LMP-1 induced expression of the anti-apoptotic gene A20 in Intestine 407 epithelial cells [169]. Similarly, LMP2A contributes to apoptosis resistance in epithelial cells. In GC cells, expression of LMP2A conferred protection against serum deprivation-induced apoptosis, a process associated with survivin overexpression [170]. Together, these findings indicate that LMP1 and LMP2A hijack host signaling pathways to suppress apoptosis and support the survival of EBV-infected epithelial cells.

#### 5.1.2. Epstein–Barr Nuclear Antigen 1 (EBNA1)

Although EBNA1 is primarily involved in maintenance of the EBV episome and regulation of viral replication, accumulating evidence also implicates this viral protein in apoptosis inhibition through modulation of oxidative stress responses and p53 signaling [171,172]. In MKN-45 gastric adenocarcinoma cells, ectopic expression of EBNA1 induced overexpression of several p53-inhibitory genes, including *MDM4*, *MDM2*, and *PSMD10*. Although reduced p53 mRNA levels were observed, this decrease did not reach statistical significance (*p* = 0.057) [173]. These findings suggest alterations in regulators of the p53 pathway but do not provide conclusive evidence of direct p53 suppression in this model.

Additional studies support the role of EBNA1 in attenuating p53-dependent apoptosis through interactions with cellular regulatory proteins. EBNA1 and p53 have been reported to bind the same domain of ubiquitin-specific peptidase 7 (USP7), an important regulator of both p53 and Mdm2. Functional analyses demonstrated that EBNA1 binding to USP7 reduced p53 levels and attenuated apoptosis in H1299 non-small-cell lung carcinoma cells [174].

EBNA1 has also been associated with disruption of promyelocytic leukemia (PML) nuclear body function. Constitutive EBNA1 expression promoted degradation of PML proteins in CNE2 and HK1 NPC cells, leading to disruption of PML nuclear bodies. These alterations were accompanied by reduced acetylation of p53 at lysine 382 (K382), impaired DNA damage repair capacity, and increased resistance to etoposide-induced apoptosis [175]. Similar findings were subsequently reported in AGS GC cells [176]. Together, these observations indicate that, beyond its role in episomal maintenance, EBNA1 contributes to apoptosis resistance through modulation of p53 signaling and DNA damage responses.

#### 5.1.3. BHRF1 (BamHI-H Rightward Open Reading Frame 1)

*BHRF1* is an EBV lytic-phase gene product with structural and functional homology to the cellular anti-apoptotic protein Bcl-2, although it can also be expressed during latency [177,178]. As a viral Bcl-2 homolog, BHRF1 inhibits apoptosis through interactions with pro-apoptotic proteins, including Bim, Bak, and Bid, thereby preventing mitochondrial outer membrane permeabilization (MOMP) and subsequent cytochrome c release, a critical event in the intrinsic apoptotic pathway [179].

Experimental studies further support the anti-apoptotic activity of BHRF1 in epithelial and tumor cell models. In NPC cells, BHRF1 induced mitochondrial membrane permeability transition (MMPT), which was associated with increased reactive oxygen species (ROS) production, activation of mitophagy, and reduced apoptosis [180]. In CNE2 NPC cells, expression of BHRF1 protected cells from apoptosis induced by the DNA-damaging agent camptothecin [181], and similar protective effects were observed following radiation exposure [182].

Beyond its effects on mitochondrial apoptosis, BHRF1 also interferes with death receptor-mediated apoptotic signaling. Intestine 407 cells ectopically expressing BHRF1 exhibited resistance to TNF-α- and Fas-mediated apoptosis [183]. Likewise, BHRF1 protected Fas-transfected MCF breast cancer cells (MCF-Fas) from TNF- and Fas-induced apoptosis through inhibition of cPLA2 and CPP32/caspase-3-like protease activation [184].

Evidence also suggests a potential contribution of BHRF1 to therapeutic resistance. Elevated BHRF1 expression was detected in EBV-positive GC cells following treatment with the pro-apoptotic agent docetaxel, supporting a possible role for this viral protein in chemoresistance [185]. Taken together, these findings indicate that BHRF1 promotes apoptosis resistance through modulation of both mitochondrial and death receptor-associated apoptotic pathways.

#### 5.1.4. BamHI-A Rightward Frame 1 (BARF1)

BARF1 has been implicated in apoptosis resistance through modulation of the balance between pro- and anti-apoptotic proteins in epithelial cells. BARF1 has been reported to increase anti-apoptotic Bcl-2 family members (Bcl-2, Bcl-xL) and reduce pro-apoptotic Bax in transfection models, shifting the Bcl-2/Bax balance toward cell survival [159].

Experimental evidence supports this anti-apoptotic role in epithelial and GC models. Introduction of BARF1 into primary epithelial and NPC cells resulted in increased Bcl-2 expression [186]. Similarly, BARF1-expressing HaCaT cells exhibited elevated Bcl-xL levels compared with untransfected controls [187]. Consistent with these observations, BARF1 expression promoted a survival-favoring Bcl-2/Bax ratio, further supporting its role in apoptosis inhibition [188].

In GC models, BARF1-induced expression of Bcl-2 and Bcl-xL was associated with activation of mitogen-activated protein kinase (MAPK) pathways, including JNK1/2/3, p38 MAPK, and ERK1/2, together with downstream c-Jun signaling [189]. Additional evidence supporting the anti-apoptotic function of BARF1 following paclitaxel (Taxol) treatment in BARF1-expressing GC cells, where increased Bcl-2/Bax ratios and reduced nuclear fragmentation were observed [188]. These findings collectively suggest that BARF1 contributes to apoptosis resistance and may promote reduced sensitivity to apoptosis-inducing therapies in EBVaGC.

#### 5.1.5. BamHI-A Leftward Frame 1 (BALF1)

BALF1 is another EBV gene product with structural homology to Bcl-2 and Bcl-xL that complements the function of BHRF1 during the early lytic phase. BALF1 contributes to apoptosis inhibition by sequestering pro-apoptotic BH3-only proteins, thereby preserving mitochondrial integrity. Notably, sequence analyses have revealed that BALF1 shares greater similarity with the cellular antiapoptotic proteins Bcl-2 and Bcl-xL than BHRF1, supporting its classification as a functional viral Bcl-2-like protein [190].

Transfection of HeLa cells with a plasmid encoding BALF1 protected against apoptosis induced by IFN-γ in combination with an anti-Fas antibody or by the topoisomerase inhibitor camptothecin. This protective effect was associated with the inhibition of the pro-apoptotic proteins Bax and Bak [190]. Similarly, BALF1 effectively inhibited 5-FU-induced apoptosis in AGS and HGC27 GC cells. Mechanistically, BALF1 was shown to stabilize endogenous Bcl-2 by reducing its ubiquitination, thereby enhancing the antiapoptotic capacity of the host cell [191].

In addition to its prosurvival functions, BALF1 has been reported to indirectly modulate apoptosis by antagonizing the antiapoptotic activity of BHRF1, acting as a negative regulator of EBV-mediated cell survival. However, unlike cellular proapoptotic BCL-2 family members, BALF1 lacks intrinsic proapoptotic activity. Furthermore, its inhibitory effect on BHRF1 appears to be cell-type-dependent, suggesting that BALF1-mediated regulation of apoptosis is influenced by host cell-specific factors [192].

### 5.2. Role of EBV-Encoded microRNAs (miR-BARTs) in Apoptotic Regulation

EBV-encoded microRNAs (miR-BARTs) contribute to apoptosis regulation in EBV-associated epithelial malignancies by modulating the expression of both pro- and anti-apoptotic factors. Several miR-BARTs target components of the p53 and mitochondrial apoptotic pathways, thereby promoting cell survival and resistance to apoptosis-inducing stimuli.

In GC models, miR-BART5-3p exhibited pronounced anti-apoptotic activity. Transfection of SGC7901 GC cells with miR-BART5-3p significantly reduced the mRNA levels of p53, Fas, Bax, and p53-upregulated modulator of apoptosis (Puma) [193]. In the same model, miR-BART5-3p also protected cells from etoposide- and ionizing radiation-induced apoptosis through regulation of Bax, Bak, Bad, and Puma protein expression [193]. Similarly, miR-BART5 reduced Puma mRNA levels in HeLa cervical and HK1 NPC cells [194]. Although a comparable reduction in PUMA mRNA was not observed in AGS GC cells [195], decreased PUMA-β protein expression was detected in miR-BART5-positive C666-1 NPC cells, which exhibited resistance to Adriamycin treatment [194].

Other EBV miRNAs target additional components of apoptotic signaling. miR-BART4-5p reduced Bid and caspase-3 protein expression in MKN1, NUGC3, and AGS GC cells [129]. In AGS cells, miR-BART9, as well as the combined expression of miR-BART11 and miR-BART12, downregulated Bim expression [195]. Moreover, transfection of EBV-negative CNE2 NPC cells with miR-BART19-3p reduced apoptosis as measured by Annexin V/propidium iodide assays [196].

Additional anti-apoptotic effects have been reported for miR-BART1-3p and miR-BART20-5p. Luciferase reporter assays demonstrated that miR-BART1-3p specifically targets the 3′-UTR of Disabled homolog 2 (DAB2), reducing its protein expression [197]. In AGS EBV-positive GC cells, inhibition of miR-BART1-3p increased expression of PARP, Bax, and cleaved caspase-3 proteins, supporting an anti-apoptotic role for this microRNA. Likewise, miR-BART20-5p reduced Bad expression in AGS cells, resulting in decreased apoptotic cell populations measured by PE-Annexin V staining and sub-G1 nuclear fragmentation analyses [198]. Notably, suppression of Bad by miR-BART20-5p also conferred resistance to 5-fluorouracil (5-FU)-induced apoptosis in AGS cells [198].

**Figure 3:**
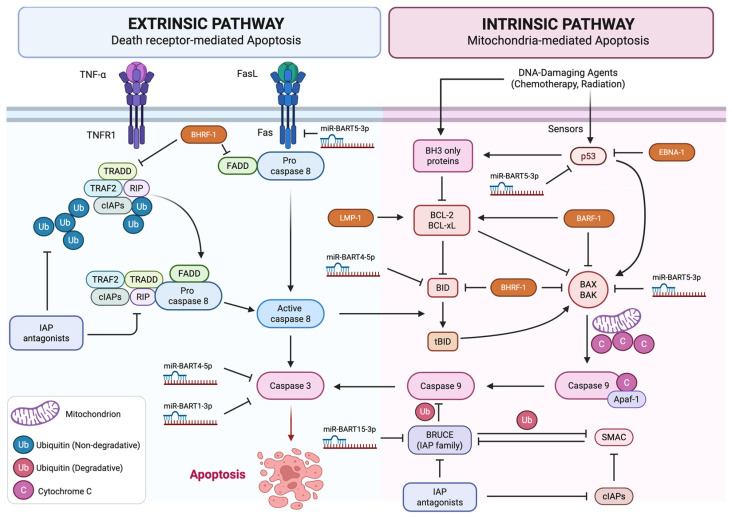
EBV modulation of apoptotic pathways. EBV factors modulate apoptosis at multiple points. In the extrinsic pathway, BHRF1 and miR-BART5-3p modulate death receptor signaling by targeting TRADD and FADD, respectively. In addition, miR-BART4-5p and miR-BART1-3p contribute to apoptosis resistance by suppressing caspase-3 activation. In the intrinsic pathway, LMP1, BARF1, EBNA1, BHRF1 and miR-BART5-3p regulate mitochondrial apoptosis by targeting BCL-2 family members, p53, BID and BAX/BAK, thereby influencing cytochrome c release. Although miR-BART15-3p can inhibit BRUCE, a member of the IAP family, EBV miRNAs collectively exert predominantly anti-apoptotic effects. Arrows indicate activation and blunt-ended lines indicate inhibition. Created in BioRender. Munoz, J.P. (2026) https://BioRender.com/me4mics.

In contrast to the predominantly anti-apoptotic activities described for most miR-BARTs, miR-BART15-3p has been associated with pro-apoptotic effects in AGS GC cells [199,200]. miR-BART15-3p increased sensitivity to 5-FU treatment through targeting of Tax1-binding protein 1 (TAX1BP1) [200], a molecule recently associated with inhibition of virus-induced apoptosis [201]. Increased apoptotic ratios were also observed in AGS cells transfected with miR-BART15-3p, an effect associated with reduced expression of baculovirus repeat-containing ubiquitin-conjugating enzyme (Bruce) [199]. Bruce (Birc6, Apollon) belongs to the inhibitor of apoptosis protein (IAP) family and promotes proteasomal degradation of caspase-9 and Smac through ubiquitination [202]. Despite these findings, the role of miR-BART15-3p in EBV-infected epithelial cells remains incompletely understood. This is relevant because EBV miRNAs collectively have been associated with anti-apoptotic effects, including reduced levels of cleaved PARP and caspase-3 in the same AGS cell model [195]. Together, these observations indicate that EBV-encoded proteins and microRNAs modulate multiple apoptotic checkpoints in both intrinsic and extrinsic apoptotic pathways (Figure 3).

Collectively, these observations indicate that EBV-encoded miRNAs modulate multiple apoptotic checkpoints and contribute to apoptosis resistance in epithelial malignancies, although individual miRNAs may exert distinct or context-dependent effects. While these findings provide mechanistic support for the role of EBV miRNAs in apoptosis regulation, many are based on in vitro experimental systems and therefore should be interpreted as evidence of biological plausibility rather than definitive proof of physiological relevance in human tumors. Table 2 summarizes the anti-apoptotic roles of EBV-encoded products (proteins and miR-BARTs) with validated targets in human epithelial cells.

**Figure 4:**
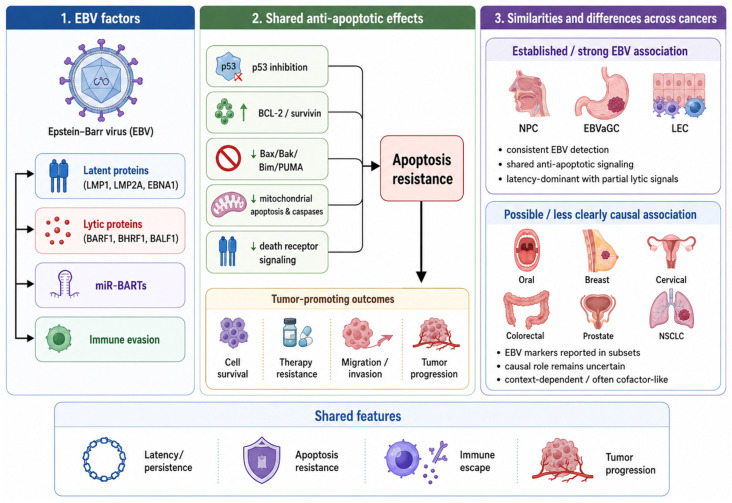
Integrated model of EBV-driven apoptosis resistance and tumorigenesis across epithelial cancers. EBV latent proteins, lytic proteins, and viral miRNAs converge on common anti-apoptotic mechanisms, including p53 inhibition, upregulation of pro-survival proteins, suppression of mitochondrial apoptosis, attenuation of death receptor signaling, and immune evasion. These mechanisms promote cell survival, therapy resistance, migration/invasion, and tumor progression. Also, it summarizes similarities and differences between tumors with strong EBV association and tumors in which EBV has been detected, but its causal role remains less clearly established.

In summary, EBV employs a coordinated network of latent proteins, lytic gene products, and viral miRNAs to modulate key apoptotic checkpoints and promote infected-cell survival. By targeting both intrinsic and extrinsic apoptotic pathways, these viral mechanisms support latency, facilitate immune evasion, and contribute to EBV-associated tumorigenesis. The effects of EBV gene products are likely to be context-dependent. Differences in latency programs, epithelial cell type, and the tumor microenvironment may influence the expression and functional consequences of these viral factors. Consequently, the relative contribution of individual EBV products to apoptosis regulation may vary across EBV-associated epithelial malignancies. These apoptosis-regulating mechanisms not only contribute to tumor development and persistence but also provide potential therapeutic targets for the treatment of EBV-associated epithelial cancers. An integrated overview of the shared and tumor type-specific mechanisms by which EBV products contribute to apoptosis resistance is shown in Figure 4.

## 6. Therapeutic Strategies Targeting EBV and Apoptotic Pathways in EBV-Associated Epithelial Tumors

The present section summarizes current and emerging therapeutic strategies aimed at restoring apoptotic signaling, targeting EBV gene products, and enhancing antitumor immune responses.

### 6.1. Apoptosis Resistance as a Therapeutic Challenge in EBV-Associated Epithelial Tumors

Because chemotherapy and radiotherapy largely kill tumor cells by activating apoptosis, the apoptotic suppression characteristic of EBV-associated epithelial malignancies can undermine their efficacy. Upregulation of anti-apoptotic proteins, blocked caspase activation, and impaired death receptor signaling jointly reduce sensitivity to DNA-damaging agents and immune-mediated cytotoxicity [203,204]. Consistent with this, high LMP1 expression has been linked to cisplatin resistance in NPC cells [205] and BARF1 expression to limited therapeutic responsiveness in gastric tumor cells [206]. Apoptosis evasion may also promote immune escape by reducing danger-associated signals and limiting antigen presentation [207,208,209]. These challenges have motivated strategies that restore apoptotic competence by modulating host survival pathways, targeting EBV gene products, and inducing antitumor immunity.

### 6.2. Modulation of Host Apoptotic Pathways and EBV Gene Products

Among host-directed approaches, BH3 mimetics are promising: they mimic BH3-only proteins and neutralize anti-apoptotic Bcl-2 family members, releasing Bax and Bak to trigger apoptosis [210]. Agents such as venetoclax and navitoclax are therefore being explored as adjuvants to sensitize tumor cells to apoptosis [211,212].

Histone deacetylase inhibitors (HDACis) also show pro-apoptotic activity by increasing histone acetylation and modulating apoptosis-related gene expression. Vorinostat (SAHA), an FDA-approved HDAC inhibitor for cutaneous T-cell lymphoma, induced EBV lytic reactivation and apoptosis in EBV-positive NPC cells, with increased PARP cleavage and activation of caspase-3, caspase-7, and caspase-9 [213,214]. Combining SAHA with bortezomib enhanced early and late apoptosis beyond either agent alone, accompanied by greater PARP and caspase cleavage, elevated ROS production, and caspase-8-dependent histone acetylation [215]. RNA-based strategies against oncogenic EBV miRNAs have likewise been proposed to restore apoptosis [216,217].

### 6.3. Targeting EBV Latency-Associated Gene Products

Targeting latency-associated genes has shown clear pro-apoptotic potential in epithelial tumors. CRISPR/Cas9 targeting of EBNA1, OriP, and W-repeat elements reduced viral load in C666-1 NPC cells, and OriP targeting sensitized cells to the DNA-damaging agents cisplatin and 5-fluorouracil (5-FU) [218]. Lentiviral RNA interference against EBNA1 induced apoptosis in EBV-positive GT-38 GC cells and inhibited tumor growth in vivo [219]. Baicalein and triptolide modulated EBNA1 expression and promoted apoptosis through increased p53 and caspase activation and downregulation of anti-apoptotic proteins [220,221,222]. The EBNA1 inhibitor VK-2019 reduced viral DNA copy number and altered apoptosis- and cell-cycle-related pathways in preclinical studies [223], with acceptable safety and on-target activity in a Phase I study of advanced EBV-positive NPC [224].

LMP1-directed approaches are also promising. In C666-1 NPC cells, LMP1 siRNA enhanced sensitivity to bleomycin and cisplatin and promoted apoptosis, reflected by PARP and caspase-3 cleavage and a trend toward increased Bax [225]. Mechanistically, LMP1 upregulates hexokinase II (HK2), a metabolic adaptation associated with apoptosis resistance [226]; HK2 binds the voltage-dependent anion channel (VDAC) at the mitochondrial outer membrane, preventing cytochrome c release and suppressing caspase-9-mediated apoptosis [227,228]. Accordingly, HK2 downregulation after LMP1 siRNA enhanced caspase-9 and PARP cleavage [226]. LMP1-targeted DNAzymes—catalytic DNA molecules that selectively cleave target RNA—reduced Bcl-2, increased caspase-3 and caspase-9 activity, promoted apoptosis, and inhibited tumor growth in vivo in NPC models, with further enhancement when combined with radiotherapy [229,230]. A randomized Phase I/II trial of the LMP1-targeted DNAzyme DZ1 with radiotherapy in NPC patients reported effects on angiogenesis and microvascular permeability, suggesting a potential radiosensitizing effect [231].

### 6.4. Immunotherapeutic Strategies in EBV-Associated Epithelial Tumors

#### 6.4.1. Therapeutic Vaccines Targeting EBV Latency Antigens

Vaccines against latent antigens have shown encouraging immunogenicity in early-phase trials. The MVA-EL vaccine (EBNA1 and LMP2) was well tolerated and induced EBV-specific T-cell responses in patients with NPC and other EBV-positive malignancies [232,233]. AdE1-LMPpoly immunotherapy (EBNA1, LMP1, and LMP2) enhanced EBV-specific responses and yielded encouraging survival outcomes in recurrent or metastatic NPC [234], supporting latency-directed vaccination for tumor control.

#### 6.4.2. Adoptive Transfer of EBV-Specific Cytotoxic T Lymphocytes

NPC expresses a restricted set of latent antigens—EBNA1, LMP1, and LMP2—capable of eliciting antigen-specific cytotoxic T-cell responses [235]. Accordingly, EBV-specific cytotoxic T-lymphocyte (EBV-CTL) therapy has shown a favorable safety profile and antitumor activity in early-phase studies of locoregional or recurrent/metastatic NPC [236,237]. However, a recent international randomized Phase III trial found that adding EBV-CTLs to gemcitabine/carboplatin did not improve overall survival or other major outcomes, although safety remained favorable [238].

#### 6.4.3. Engineered T-Cell Therapies

Genetically engineered T cells are also in development. HLA-matched EBV-specific T-cell receptor (TCR-T) cells can be rapidly generated against latent antigens; preclinically, LMP2-specific TCR-T cells showed potent antigen-specific cytotoxicity and suppressed NPC growth in vivo [239], prompting a Phase I trial of LMP2-specific IL-12-secreting TCR-T cells in EBV-positive metastatic or refractory NPC [240]. Chimeric antigen receptor (CAR)-T approaches are likewise advancing, with LMP1 a target of particular interest: LMP1-specific CAR-T cells eliminated LMP1-positive cells in vitro and inhibited LMP1-expressing NPC xenografts in vivo [241], and a clinical trial has been registered to assess their safety and preliminary efficacy in NPC [242].

#### 6.4.4. Bispecific Antibody-Based Immunotherapy

Antibody-based approaches can selectively target EBV-associated epithelial tumors. TCR-like bispecific antibodies recognizing LMP2A peptide–HLA complexes showed potent antitumor activity and favorable safety in preclinical models [243]. A bispecific antibody–drug conjugate co-targeting the EBV antigen GP220/350 and EGFR outperformed conventional EGFR-targeted therapy in preclinical EBV-positive NPC, supporting dual targeting of viral and tumor-associated antigens to improve selectivity and efficacy [244].

### 6.5. EBV Lytic Induction Strategies in Epithelial Tumors

An alternative strategy exploits lytic reactivation to sensitize tumor cells to antiviral and pro-apoptotic therapies. Cytolytic virus activation (CLVA) combines lytic inducers (gemcitabine, valproic acid) with antivirals (ganciclovir or valganciclovir); chemotherapy-induced lytic activation sensitized EBV-positive epithelial tumor cells to ganciclovir-mediated killing [245]. Translational studies in EBVaGC and a pilot observation in three patients with refractory NPC support this rationale, with treated patients showing rising circulating EBV DNA, consistent with release from apoptotic tumor cells, and disease stabilization [246,247,248]. Although clinical experience remains limited, these findings warrant further evaluation of lytic induction in EBV-associated epithelial malignancies.

Together, these findings indicate that apoptosis can be restored in EBV-associated epithelial malignancies through complementary strategies: modulating host apoptotic regulators, inhibiting EBV latency-associated gene products, enhancing EBV-specific immunity, and inducing the lytic cycle. The progression of several approaches from preclinical models to early-phase clinical studies highlights their potential to overcome viral-mediated survival signaling and improve therapeutic responses. A summary is provided in Table 3.

## 7. Conclusions

EBV has evolved a coordinated network of latent and lytic gene products that enables long-term persistence within host cells while promoting resistance to programmed cell death. This ability to manipulate apoptotic signaling represents a central mechanism underlying both viral latency and EBV-associated epithelial oncogenesis. Through viral proteins and non-coding RNAs, EBV modulates multiple checkpoints of the intrinsic and extrinsic apoptotic pathways, thereby promoting infected-cell survival, facilitating immune evasion, and supporting malignant progression.

The evidence reviewed here indicates that EBV-associated epithelial malignancies frequently express viral products that, largely in experimental models, interfere with mitochondrial apoptosis, death receptor signaling, and p53-dependent responses. Latent proteins such as LMP1, LMP2A, and EBNA1, together with lytic proteins including BHRF1, BALF1, and BARF1, have been shown—mainly in vitro—to promote cell survival by enhancing pro-survival signaling, suppressing pro-apoptotic mediators, and impairing responses to cellular stress and DNA damage. EBV-encoded miRNAs, particularly miR-BARTs, further reinforce these effects through post-transcriptional regulation of apoptotic and immune-related targets. Collectively, these mechanisms create a cellular environment that favors viral persistence and tumor progression.

The extensive disruption of apoptotic pathways in EBV-positive epithelial tumors has important therapeutic implications. Resistance to apoptosis can limit the efficacy of chemotherapy and radiotherapy and may contribute to unfavorable clinical outcomes. Consequently, therapeutic approaches aimed at restoring apoptotic sensitivity, including BH3 mimetics, histone deacetylase inhibitors, lytic-cycle induction strategies, and EBV-targeted immunotherapeutic or gene-silencing interventions, have shown promising preclinical and early clinical activity. However, given the multifactorial nature of EBV-associated tumorigenesis, targeting apoptotic pathways alone is unlikely to be sufficient for durable therapeutic responses and should be considered within broader treatment strategies that also address viral persistence, immune evasion, and additional oncogenic signaling networks.

Nevertheless, the biological impact of individual viral factors may depend on the cellular and microenvironmental context, further complicating the identification of broadly effective therapeutic targets. Moreover, tumor cells may activate compensatory survival pathways that reduce the effectiveness of apoptosis-directed therapies. The lack of validated predictive biomarkers limited clinical evidence for several emerging approaches, and concerns regarding off-target toxicity further complicate patient selection and therapeutic decision-making. These limitations underscore the need for improved biomarkers, rational combination strategies, and prospective clinical studies.

Although significant progress has been made in elucidating the mechanisms by which EBV dysregulates apoptosis, further validation of therapeutic targets, predictive biomarkers, and combination approaches will be required to establish their clinical utility in EBV-associated epithelial malignancies. Continued integration of molecular, translational, and clinical research will be essential to translate these mechanistic insights into more effective therapies and improved patient outcomes.

## 8. Future Directions

Although numerous EBV gene products have been implicated in apoptosis resistance, important questions remain regarding their relative contribution to epithelial tumorigenesis. Most studies have evaluated individual viral proteins or miRNAs in isolation, whereas EBV-associated tumors simultaneously express multiple viral products. Future studies should therefore address how latent proteins, lytic proteins, and miR-BARTs cooperate or potentially counteract one another within the same cellular context.

Another unresolved issue concerns the context-dependent effects of EBV-encoded miRNAs. While most miR-BARTs have been associated with anti-apoptotic functions, miR-BART15-3p has demonstrated pro-apoptotic activity in some experimental models. Whether these apparently opposing effects reflect differences in cell type, viral latency pattern, target gene availability, or experimental conditions remains unclear and warrants further investigation.

The relative importance of latent and lytic gene products in apoptosis regulation also remains incompletely understood. Although proteins such as LMP1, LMP2A, and EBNA1 are frequently studied because of their sustained expression in epithelial tumors, increasing evidence suggests that lytic proteins, including BHRF1 and BARF1, may contribute substantially to apoptosis resistance. Defining the temporal and functional interactions between latent and lytic programs represents an important priority for future research.

Finally, much of the current evidence is derived from established cell lines and experimental overexpression systems. Validation of these findings in primary tumor samples, patient-derived models, and clinically annotated cohorts will be necessary to determine the biological and clinical relevance of EBV-mediated apoptosis regulation in epithelial malignancies.

## Figures and Tables

**Table 1:** Expression profiles of EBV-encoded proteins and microRNAs in EBV-associated epithelial tumors.

Tumor Type	EBV Gene Expression (mRNA/Protein)	Main Finding/Associated Phenotype	Refs.
Nasopharyngeal carcinoma	EBER1, EBER2, EBNA1, EBNA2, EBNA3A, EBNA3B, and EBNA3C	RNA-seq profiling showed that EBV gene expression in NPC is not limited to classical latency II, but may include type III latency genes and several lytic transcripts. EBV expression patterns were associated with signaling pathways related to inflammation, protein metabolism, and ECM-receptor interaction.	[120]
	BZLF1, BRLF1, BALF1, BARF1, BMRF1, and BLLF1	These studies support the presence of limited or partial lytic gene expression in NPC biopsies. BARF1 was reported in NPC tissues, and *BALF1* transcripts were also detected in NPC biopsies.	[121,122,123,124]
	miR-BARTs	EBV-encoded BART miRNAs are highly expressed in primary NPC tumors, supporting their relevance as post-transcriptional regulators.	[125]
Gastric adenocarcinoma	EBER1, EBNA1, and LMP2A	EBV-associated gastric carcinoma shows latent EBV gene expression, supporting EBVaGC as a distinct EBV-positive gastric cancer subset.	[126]
	BHRF1, BZLF1, BRLF1, BALF1, and BARF1	EBVaGC samples may show expression of both latent and lytic EBV genes, suggesting that partial lytic activation can occur in EBV-positive gastric tumors.	[126,127,128]
	miR-BARTs	EBV miRNAs are highly expressed in EBVaGC and are linked to cancer-related pathways, particularly apoptosis regulation; reduced apoptosis and Bid downregulation were reported in EBVaGC-related models/tissues.	[129]
LEC	EBERs, EBNA1, and LMP1	EBV latent gene expression supports the close association between EBV infection and lymphoepithelial carcinoma, particularly in sinonasal LEC.	[130]
	BZLF1	Detection of *BZLF1* suggests that partial lytic activation may also occur in EBV-positive LEC.	[130]
	miR-BART5-3p and miR-BART20-3p	Integrated genomic analysis of pulmonary LEC identified EBV-related molecular features, including miR-BARTs, and suggested that these viral miRNAs may contribute to the distinct molecular signature of pLELC.	[131]
Oral carcinoma	EBER1, EBNA1, EBNA2, EBNA3B, EBNA3C, LMP1, LMP2A, and LMP2B	EBV gene expression has been detected in oral carcinoma/OSCC. Some studies suggest EBV may be associated with oral carcinogenesis, although causality remains uncertain.	[132,133,134,135]
	BZLF1 and BMRF2	Detection of lytic-associated EBV genes suggests possible EBV reactivation or partial lytic activity in a subset of oral carcinoma tissues.	[134,135]
Breast cancer	EBER1, EBER2, EBNA1, EBNA2, EBNA3B, LMP1, and LMP2A	EBV has been detected in subsets of breast carcinoma samples, but the association remains controversial. Some studies reported EBV expression in epithelial tumor cells, whereas others found no strong clinicopathological association.	[136,137,138,139,140]
Cervical carcinoma	EBER1, EBNA1, EBNA2, and LMP1	EBV genes have been detected in CIN and invasive cervical carcinoma, often in the context of HPV co-infection. The studies support a possible cofactor role, but not definitive causality.	[141,142,143,144,145]
	BARF1	BARF1 expression was reported in cervical lesions with HR-HPV/EBV co-presence. Experimental BARF1 expression was also used to evaluate phenotypic changes in cervical cancer cells.	[146]
Colorectal cancer	EBERs, EBNA1, and LMP1	EBV markers have been detected in colorectal carcinoma, and one cited study specifically evaluated EBV in relation to Fascin expression, suggesting a possible association with tumor-related phenotypes.	[147,148]
Prostatic adenocarcinoma	EBER1, EBER2, EBNA1, EBNA2B, EBNA3A, EBNA3B, EBNA3C, EBNALP, LMP1, and LMP2A	EBV-positive prostate carcinoma samples showed latency II/III-like expression profiles. Later work evaluated EBV-positive versus EBV-negative tumors for markers of aggressiveness and survival-related differences.	[118,149]
	BZLF1 and BHRF1	Detection of lytic-associated genes suggests that EBV-positive prostate carcinoma may include both latent and lytic viral expression patterns.	[118]
Non-small-cell lung cancer	EBER1, EBNA1, and LMP1	EBV latent markers have been reported in lung cancer, but detection rates vary across studies. Some reports associate EBV with poorly differentiated tumors or specific lung cancer subtypes, while the overall role remains heterogeneous.	[150,151,152,153,154]
	BARF1	BARF1 was detected in a subset of EBV-positive lung carcinomas and was associated experimentally with increased migration, invasion, and EMT, suggesting a possible role in lung cancer progression.	[151]

**Table 2:** Anti-apoptotic roles of EBV-encoded products (proteins and miR-BARTs) with validated targets in human epithelial cells.

EBV Product	Target	Cell Type	Refs
	p53 USP7	MKN-45 GC cells	[173]
EBNA1	p53 USP7	H1299 NSCLC cells	[174]
	PML proteins	CNE2 and HK1 NPC cells	[175,176]
	PML proteins	AGS GC cells	[175,176]
LMP1	Bcl-2	HONE-1, NPC-TW01, and NPC-TW04 NPC cells	[167]
	Survivin	CNE1 NPC cells	[168]
	A20	Intestine 407 epithelial cells	[169]
LMP2	Survivin	MKN-1 GC cells	[170]
BHRF1	cPLA2 and CPP32/caspase-3-like protease	MCF-Fas BC cells	[184]
BALF1	Bak and Bax	HeLa cells	[190]
BARF1	Bcl-2	NP69 NPC cells	[186]
	Bcl-2 and Bcl-xL	MKN28, SGC7901, and BGC823 GC cells	[189]
miR-BART5-3p	p53, Fas, Bax, Bak, Bad, and Puma	SGC7901 GC cells	[193]
miR-BART4-5p	Bid and Caspase-3	MKN1, NUGC3, and AGS GC cells	[129]
miR-BART9, miR-BART11, miR-BART12	Bim	AGS GC cells	[195]
miR-BART5	Puma	HeLa cervical cells and HK1 NPC cells	[194]
miR-BART1-3p	DAB2	AGS GC cells	[197]
miR-BART20-5p	Bad	AGS GC cells	[198]

**Table 3:** Summary of therapeutic strategies targeting EBV and apoptotic pathways in EBV-associated epithelial tumors.

Strategy	Target	Pro-Apoptotic Mechanism	Examples	Development Stage	Refs.
BH3 mimetics	Bcl-2 family proteins	Release Bax/Bak and activate mitochondrial apoptosis	Venetoclax and Navitoclax	Preclinical	[211,212]
HDAC inhibitors	Epigenetic regulators	Induce pro-apoptotic genes, caspase activation, and EBV lytic reactivation	Vorinostat (SAHA) and SAHA + bortezomib	Preclinical	[213,214,215]
EBNA1-targeted therapies	EBNA1	Induce p53 and caspase activation; reduce survival signaling	RNAi, baicalein, triptolide, VK-2019	Preclinical; Phase I	[219]
LMP1 silencing	LMP1	Increase caspase activation and reduce HK2-mediated apoptosis resistance	LMP1 siRNA	Preclinical	[226]
DNAzymes	LMP1 mRNA	Cleave target RNA, reduce Bcl-2, and activate caspases	DZ1	Preclinical; Phase I/II	[229,230,231]
Therapeutic vaccines	EBNA1, LMP1, LMP2	Enhance EBV-specific T-cell responses	MVA-EL and AdE1-LMPpoly	Phase I	[232,233]
EBV-specific CTLs	EBV latent antigens	Antigen-specific killing of tumor cells	EBV-CTLs	Early clinical; Phase III completed	[236,237,238,243]
TCR-T cells	LMP2	Redirect T cells against EBV-positive tumors	LMP2-specific TCR-T	Preclinical; Phase I	[239,240]
CAR-T cells	LMP1	Antigen-directed tumor cell killing	LMP1-specific CAR-T	Preclinical; clinical trial ongoing	[241,242]
Bispecific antibodies	LMP2A peptide–HLA; GP220/350 and EGFR	Enhance selective tumor cell killing	TCR-like bispecific antibodies; GP220/350-EGFR ADC	Preclinical	[244]
Lytic induction therapy	EBV lytic cycle	Sensitize tumor cells to antiviral and apoptotic therapies	CLVA	Preclinical; limited clinical evidence	[246,247,248]

## Data Availability

Data sharing is not applicable. No new data were created or analyzed in this study.

## Abbreviations

The following abbreviations are used in this manuscript:

5-FU	5-fluorouracil
AIF	Apoptosis-inducing factor
Akt	Protein kinase B
Apaf-1	Apoptotic protease-activating factor 1
ATP	Adenosine triphosphate
Bak	Bcl-2 homologous antagonist/killer
BALF1	BamHI-A leftward open reading frame 1
BALF5	Epstein–Barr virus DNA polymerase catalytic subunit
BARF1	BamHI-A rightward frame 1
BART	BamHI-A rightward transcript
Bax	Bcl-2-associated X protein
Bcl-2	B-cell lymphoma 2
Bcl-xL	B-cell lymphoma-extra large
BHRF1	BamHI-H rightward open reading frame 1
Bid	BH3-interacting domain death agonist
Bim	Bcl-2-interacting mediator of cell death
BMRF1	Epstein–Barr virus polymerase processivity factor
BMRF2	Epstein–Barr virus glycoprotein BMRF2
BRLF1	BamHI-R rightward open reading frame 1
BZLF1	BamHI-Z leftward open reading frame 1
Cas9	CRISPR-associated protein 9
CD	Cluster of differentiation
CI	Confidence interval
CLVA	Cytolytic virus activation
CRISPR	Clustered regularly interspaced short palindromic repeats
DAB2	Disabled homolog 2
DED	Death effector domain
DISC	Death-inducing signaling complex
EBNA	Epstein–Barr nuclear antigen
EBNA-LP	Epstein–Barr nuclear antigen leader protein
EBER	Epstein–Barr virus-encoded small RNA
EBV	Epstein–Barr virus
EBVaGC	Epstein–Barr virus-associated gastric carcinoma
ERK	Extracellular signal-regulated kinase
FADD	Fas-associated death domain protein
FasL	Fas ligand
FLICE	FADD-like interleukin-1β-converting enzyme
GC	Gastric carcinoma
HDAC	Histone deacetylase
HDACi	Histone deacetylase inhibitor
HPV	Human papillomavirus
HSIL	High-grade squamous intraepithelial lesion
IAP	Inhibitor of apoptosis protein
IE	Immediate-early
JAK	Janus kinase
JNK	c-Jun N-terminal kinase
Keap1	Kelch-like ECH-associated protein 1
LEC	Lymphoepithelial carcinoma
LMP	Latent membrane protein
LSIL	Low-grade squamous intraepithelial lesion
MAPK	Mitogen-activated protein kinase
Mcl-1	Myeloid cell leukemia 1
MHC	Major histocompatibility complex
miRNA	MicroRNA
miR-BART	Epstein–Barr virus-encoded BART microRNA
miR-BARTs	Epstein–Barr virus-encoded BART microRNAs
MOMP	Mitochondrial outer membrane permeabilization
MMPT	Mitochondrial membrane permeability transition
NF-κB	Nuclear factor kappa B
NPC	Nasopharyngeal carcinoma
Nrf2	Nuclear factor erythroid 2-related factor 2
NSCLC	Non-small-cell lung cancer
OR	Odds ratio
OriLyt	Lytic origin of replication
OriP	Origin of plasmid replication
OSCC	Oral squamous cell carcinoma
PARP	Poly(ADP-ribose) polymerase
Par-4	Prostate apoptosis response-4
PE	Phycoerythrin
PI3K	Phosphoinositide 3-kinase
PML	Promyelocytic leukemia protein
PSMD10	Proteasome 26S subunit, non-ATPase 10
PUMA	p53-upregulated modulator of apoptosis
RNAi	RNA interference
ROS	Reactive oxygen species
SAHA	Suberoylanilide hydroxamic acid
SCLC	Small-cell lung cancer
STAT	Signal transducer and activator of transcription
TAX1BP1	Tax1-binding protein 1
tBid	Truncated Bid
TNF-α	Tumor necrosis factor alpha
TNFR	Tumor necrosis factor receptor
TNFR1	Tumor necrosis factor receptor 1
TPA	12-O-tetradecanoylphorbol-13-acetate
TRADD	TNFR1-associated death domain protein
TRAIL	TNF-related apoptosis-inducing ligand
UTR	Untranslated region
USP7	Ubiquitin-specific peptidase 7
XIAP	X-linked inhibitor of apoptosis protein
